# Reducing Perioperative Anxiety and Postoperative Discomfort in Children With Hypnosis Before Tonsillotomy and Adenoidectomy: A Prospective Randomized Trial

**DOI:** 10.1002/hsr2.70484

**Published:** 2025-03-09

**Authors:** Barbara Schmidt, Claudia Thomas, Antje Göttermann, Winfried Meißner, Katharina Geißler, Orlando Guntinas‐Lichius, Anne Schirrmeister

**Affiliations:** ^1^ Institute for Psychosocial Medicine, Psychotherapy and Psychooncology Jena University Hospital Jena Germany; ^2^ Clinic for Anesthesiology and Intensive Care Medicine Jena University Hospital Jena Germany; ^3^ Department of Otorhinolaryngology Jena University Hospital Jena Germany

**Keywords:** anxiety, children, hypnosis, surgery

## Abstract

**Background and Aims:**

Children undergoing tonsillotomy (TT) or adenoidectomy (AT) often suffer from anxiety before and pain or nausea afterward. Greater preoperative anxiety in children and their parents is associated with increased postoperative discomfort. The aim of our study is to test if a preoperative hypnosis intervention reduces perioperative anxiety and thereby alleviates postoperative discomfort.

**Methods:**

In a previous study, we developed a narcosis comic to reduce children's preoperative anxiety. Now, we investigate whether a hypnosis audio intervention further reduces children's perioperative anxiety. Here, a little monkey describes the surgery as an adventurous space journey. We included children 3−6 years old receiving TT or AT. Thirty‐four children prepared for the surgery with the hypnosis audio intervention in addition to the comic (comic+hypnosis group), while 30 children received the comic only (comic group). All children received preoperative sedation via midazolam. We measured children's subjective well‐being before and after surgery, parents' anxiety before surgery, children's anxiety during surgery, and children's postoperative pain.

**Results:**

Children showed high well‐being before and after surgery in both groups with subjective distress ratings around 2 out of 10. Parents' anxiety was on a moderate level in both groups with anxiety ratings around 42 on the STAI‐S scale from 20 to 80. Children's anxiety was low to moderate in both groups during surgery with mYPAS ratings of 33 on a scale from 20 to 100. In the postoperative telephone interviews, children reported medium pain ratings with maximum pain values around 5 out of 10 in both groups with no significant differences in any postoperative outcome between groups.

**Conclusion:**

Our study shows that all children participating in our study reported high well‐being and low anxiety. In future studies, it should be assessed if the combination of nonmedical interventions like narcosis comic and hypnosis shows an additive effect in non‐medicated children.

## 1 Introduction

Acute stressful situations often occur in clinical settings, for example, when patients are facing surgery. The younger the patients, the more anxious they are. Preoperative anxiety is very common for children with incidence rates up to 75% [1]. Especially young children do not understand all the details of why and how the surgery takes place. Preoperative anxiety in children is associated with a higher incidence of negative medical outcomes like postoperative pain and nausea and even long‐term behavioral effects with symptoms of posttraumatic stress disorders [2, 3]. Another important predictor for preoperative anxiety and postoperative pain and stress symptoms in children is parents' anxiety [3]. It is common that children with severe preoperative anxiety suffer from nightmares, separation anxiety, eating disorders, and fear of doctors for a year after surgery [3]. Anxious children have more pain than less anxious children both during and 3 days after tonsillectomies and adenoidectomies [2].

One way to reduce preoperative anxiety in children undergoing surgeries is to provide information that explains the why and how of the medical procedures [4]. Providing child‐friendly information is central to Enhanced Recovery After Surgery (ERAS) protocols and can reduce children's preoperative anxiety, postoperative pain, and postoperative opioid consumption [5, 6]. Earlier, at Jena University Hospital, we developed a narcosis comic with “Manchu the monkey” as the hero leading children through the medical procedures of the surgery. Children receive this comic routinely during the pre‐anesthetic examination before their surgery.

Hypnosis can prevent preoperative anxiety in children [4]. Hypnosis is particularly effective in treating anxiety and stress [7, 8, 9, 10], and children are very receptive to hypnosis [11]. The effect of hypnosis on negative affect and pain during surgery is generally very large [12, 13, 14, 15, 16, 17]. Suggestions contain an imagined positive outcome of surgery, which significantly reduced postoperative pain ratings and significantly shortened the overall hospital stay [18]. Previous evidence shows that a hypnosis intervention before surgery is more effective in reducing preoperative anxiety and postoperative behavioral disorders than midazolam, a common benzodiazepine medication to reduce anxiety before surgeries [19].

In our current study, we investigate the effectiveness of a hypnotherapeutic audio intervention in addition to a narcosis comic before tonsillotomies and adenoidectomies in 3−6‐year‐old children, using self and peer assessment instruments. The audio intervention is available in German language at https://manchu.uni-jena.de/Home/Index?code=8bfa9aea-a744-447f-9ab1-0c9edb364915.

We included 3–6‐year‐old children scheduled for tonsillotomy (TT) or adenoidectomy (AT) in the Department of Otorhinolaryngology at the Jena University Hospital. These surgeries belong to the most frequent surgeries performed in children [20]. Children in the comic+hypnosis group received a hypnosis audio intervention in addition to the narcosis comic. Children in the comic group only received the narcosis comic, which is part of the standard treatment. The hypnosis intervention picks up on the contents of the comic and suggests a positive outcome of the surgery. Hypnotherapeutic interventions via an audio file before surgery are similarly effective as interventions with a hypnotherapist present [13]. All children received midazolam in a dosage adjusted to their weight as premedication (mean dosage: 7.9 mg, SD = 1.4 mg).

Our primary outcome was the self‐rated distress of children before and after surgery, measured via the subjective unit of distress (SUD) scale [21]. The secondary outcome was children's postoperative well‐being measured with the QUIPS‐infant quality analysis questionnaire (QUIPS for “Qualitätsverbesserung in der postoperativen Schmerztherapie,” which means quality improvement in postoperative pain therapy [22]). In the QUIPS‐infant, children rate their pain using the validated faces scale according to Hicks et al. [23], as well as their fatigue and nausea in a telephone call 1 day after surgery.

## 2 Methods

### 2.1 Trial Design

We used a parallel design with an experimental group (comic+hypnosis), receiving a narcosis comic and a hypnosis audio intervention before surgery and a control (comic) group only receiving the narcosis comic before surgery. Children were randomly assigned to the comic+hypnosis or comic group after informed consent had been given during the pre‐anesthetic examination, which took place at least 1 day before surgery. All children received the comic, and the children in the comic+hypnosis group also received a personalized link to the audio intervention with instructions to listen to it as often as desired before surgery. During the pre‐anesthetic examination, children also indicated how distressed they felt on a SUD scale. Before surgery, the mYPAS‐SF was completed by the attending physician in the operating lock and once the mask was put on. One day after surgery, we called children's parents and used the QUIPS‐infant used to assess postoperative pain, nausea, and vomiting. Once again, the SUD scale was used by the children to evaluate their condition.

The local ethics committee at the Jena University Hospital approved the study (2020‐2036_1‐BO). We also preregistered the study at the DRKS (German Register for Clinical Studies), available via https://drks.de/search/de/trial/DRKS00031599. We followed the CONSORT statement for randomized trials.

### 2.2 Patients

We included children undergoing TT or AT aged 3−6 years at the Jena University Hospital. We excluded children with non‐sufficient German language skills as both the narcosis comic and the hypnosis intervention were in the German language. The Department of Otorhinolaryngology, Jena University Hospital, selected eligible children who were scheduled for TT or AT for the study team.

### 2.3 Interventions

#### 2.3.1 Narcosis Comic

The comic describes all successive events that occur on the day of children's surgery. The main character in the Narcosis comic is a little monkey called Manchu. This comic was developed by coauthors Dr. Anne Schirrmeister and Dr. Claudia Thomas and drawn by the graphic artist Sandra Bach (https://www.sandruschka.de/). The comic is available via this link: https://www.uniklinikum-jena.de/kai/Patienten+und+Besucher/Narkose/Kinder/Narkosecomic.html. For more details, please see our Supporting Information Material.

### 2.4 Hypnosis Audio Intervention

We developed the hypnosis audio intervention with similar contents as the narcosis comic. The protagonist, Manchu the monkey himself, talks to the child. The audio intervention is available here https://manchu.uni-jena.de/Home/Index?code=8bfa9aea-a744-447f-9ab1-0c9edb364915 and takes 18 min. Children in the experimental group got a flyer with a QR code leading them to the hypnosis audio intervention. We developed a way to check if children listened at least once to the whole hypnosis audio intervention as we personalized the QR code for every child. For more details, please see our Supporting Information Material.

### 2.5 Outcomes

There were three main time points to measure the outcomes of the study. On the day before surgery, we obtained informed consent of parents to participate in the study and parents and children received all study materials. That was the narcosis comic, the flyer with the QR code to the hypnosis audio intervention for the experimental group and an anxiety questionnaire for the parents that they should return on the day of the surgery. As our primary outcome, we assessed children's well‐being on a SUD showing a relaxed happy face (0) on the one end of the scale and an anxious unhappy face on the other end of the scale (10) [21]. We used scales with girl faces for girls and boy faces for boys. Children were asked how they felt right now and to identify the picture that reflected their current feelings best.

On the day of the surgery, parents gave us their anxiety questionnaire (STAI‐State [24]), back that measures parents' preoperative anxiety. We measured children's anxiety with the Yale preoperative anxiety scale mYPAS‐SF (original YPAS [25]; modified short form [26]) where medical doctors rate children's behavior on two occasions: at the operating lock and when children got the mask on.

The day after surgery, we called the parents to assess our secondary outcome and asked them about their child's pain using the QUIPS‐infant pain questionnaire [22]. In the QUIPS‐infant, children rate their pain using the validated faces scale according to Hicks et al. [23] and a standardized procedural guide. Hicks et al. [23] used the faces pain scale in 4‐year‐old children and showed that most children were able to use this scale so that the results were consistent with other established pain measures. In addition, we asked children again about their feeling using the SUD showing a relaxed happy face (0) on the one end of the scale and an anxious unhappy face on the other end of the scale (10) [21]. In the comic+hypnosis group, we asked parents about their experience with the hypnosis audio intervention.

### 2.6 Sample Size

Based on the meta‐analysis by Montgomery et al. [13] on the effectiveness of hypnosis in surgical patients, we assumed an effect size of at least *d* = 0.7 for the effect of hypnotic suggestions on negative affect and pain before surgery. Using the program G*power [27], we calculated the necessary sample size to detect such an effect in a between‐groups design with an alpha level of 0.05 and a statistical power of 0.85. The necessary sample size calculated for a one‐sided test was 31 patients per group. To account for possible dropouts, we planned 40 patients per group.

### 2.7 Randomization

Children were assigned to the two groups by a predetermined order. We sorted the study material for 31 patients in the comic group and 31 patients in the comic+hypnosis group in a folder in a predetermined, randomized order. When a child came to the premedication unit and agreed to participate in the study, the child received the pack of study material that was on top of the folder. When the patients were recruited, it was not clear for the study personnel to which group they would be assigned. The medical doctor in the premedication unit handed the comic to every participating child and the individual QR code for the hypnosis intervention to the children in the comic+hypnosis group according to the study material he/she took from the folder. For the comic+hypnosis group, we checked via the individual QR code if the child listened at least once to the hypnosis audio intervention without breaks before surgery. When this was not the case (see flowchart for details), we put another pack of materials in the study material folder to replace the child in the comic+hypnosis group. The goal was to have at least 30 patients in each group, which we achieved.

### 2.8 Statistical Testing

We performed Welch two‐sample *t*‐tests that are recommended for two samples with different variances to compare the outcomes in the two groups. All tests were two‐sided. To perform our analyses, we used the statistical program R [28].

## 3 Results

### 3.1 Participant Flow

**Figure 1:**
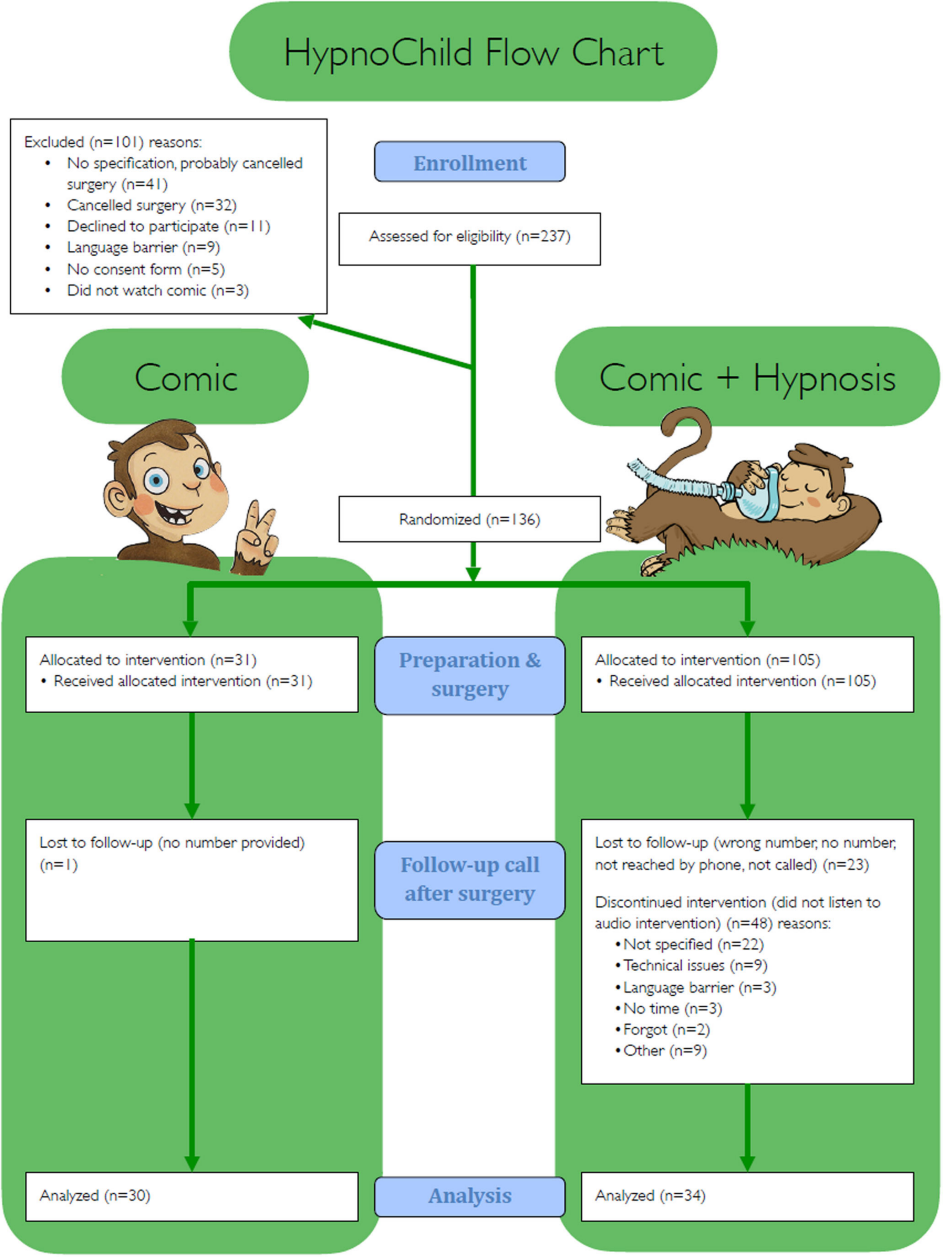
Flowchart showing the number of children who we randomly assigned to the comic or comic+hypnosis group, who received intended treatment, and were analyzed. The chart also shows the losses and exclusions after randomization, together with the reasons.

Figure 1 shows that there were 237 children initially screened for eligibility. We excluded 101 children because they (probably) canceled surgery (73 children), because their parents declined to participate (11 children), because of a language barrier (9 children), because of a lack of consent form (5 children), or because they did not watch the comic (3 children). We randomized the remaining 136 children into the comic or hypnosis+comic group. In the comic group, 31 children received and read the comic. One child was lost in the follow‐up call 1 day after surgery, so we analyzed 30 children in the comic group. In the comic+hypnosis group, 105 children received the comic and the QR code for the hypnosis audio intervention. We lost the following children in the follow‐up call due to the following reasons: we did not reach them via telephone (23 children) or they did not listen to the hypnosis audio intervention (48 children). The reasons for not listening to the hypnosis were not specified (22 children), technical issues with scanning the QR code (9 children), no time (3 children), forgot to do it (2 children), or other reasons (9 children). Therefore, we analyzed 34 children in the comic+hypnosis group.

### 3.2 Recruitment

We started recruiting in July 2021 and ended in May 2023. The follow‐up call was 1 day after surgery, so the recruitment dates are the same for follow‐up. We stopped the trial after we reached at least 30 patients in each group.

### 3.3 Baseline Data

The children in our groups were 3−6 years old. The mean age (SD) was 3.6 (0.6) years in the comic group and 4.2 (1.0) years in the comic+hypnosis group. In the comic group, we had 19 male and 11 female children; in the comic+hypnosis group, we had 17 male and 17 female children. In the comic group, 2 children had only AT, 18 children had AT with paracentesis on both sides, and 10 children had TT, AT, and paracentesis on both sides. In the comic+hypnosis group, 4 children had only AT, 1 had only TT on both sides, 23 children had AT with paracentesis on both sides, and 6 children had TT, AT, and paracentesis on both sides (Table 1).

**Table 1:** Demographic data of the sample.

	Comic group	Comic+hypnosis group
Age (years)	3.6 (0.6)	4.2 (1.0)
Male/female	19/11	17/17

### 3.4 Subjective Discomfort Rated by Children With Visual Analogue Scale

**Figure 2:**
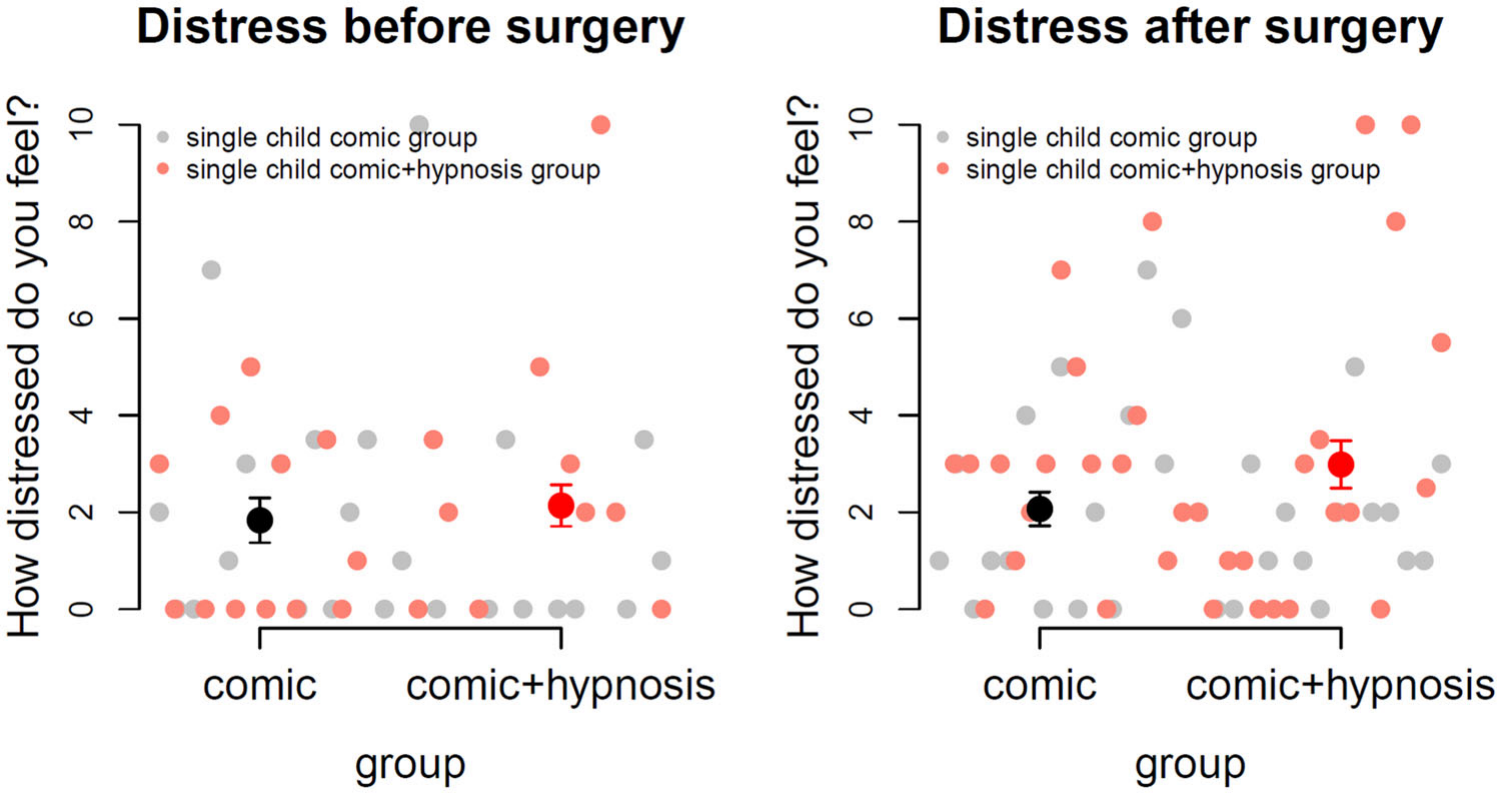
Subjective discomfort rated by children before and after surgery on a visual analogue scale. Low ratings indicate low distress. We found that all children showed low distress both before and after surgery.

Children rated their subjective discomfort on visual analogue scales showing faces. Girls had girl faces, boys had boy faces with 0 indicating low distress with a smiling face and 10 indicating high distress with a crying face. The first measurement was 1 day before surgery at the premedication unit, the second measurement on the follow‐up call 1 day after surgery. We found generally low levels of distress for all children with no significant differences between groups (before surgery: *p* = 0.68, after surgery: *p *= 0.14) and between time‐points (*p* = 0.24). Figure 2 shows the scatter plots, mean values, and standard errors of the mean for both groups and time‐points. The mean distress rating was 2.3 (SD = 2.5), indicating low distress in children before and after surgery.

### 3.5 Parents' Anxiety Before Surgery

**Figure 3:**
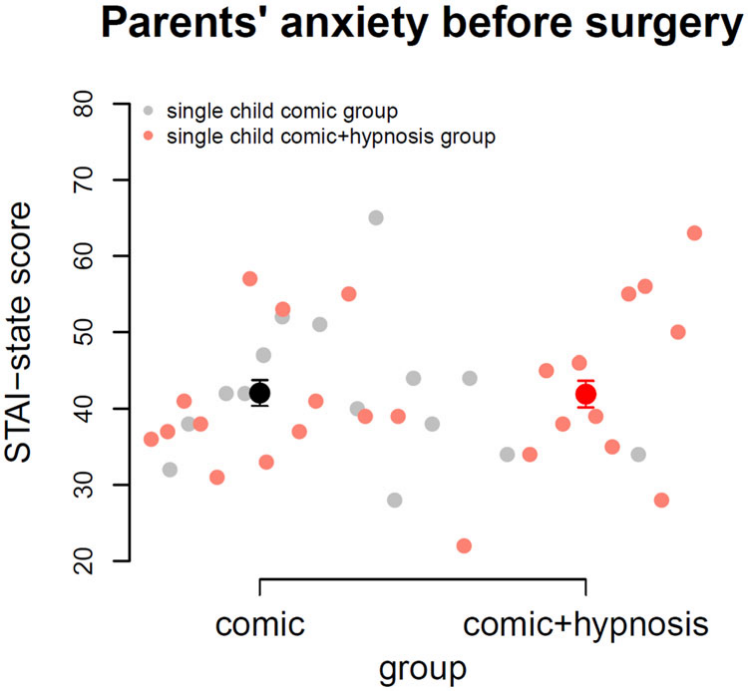
Parents' anxiety before surgery, measured with the state‐trait anxiety inventory (STAI)‐state, showed no group differences and generally moderate levels of anxiety.

We measured parents' anxiety with the STAI‐State [24]. Lowest anxiety equals a sum score of 20, highest anxiety a sum score of 80. We found no significant difference between groups (*p *= 0.96). Mean anxiety score was 42, indicating moderate anxiety in parents before surgery. Figure 3 shows the scatter plots of both groups with mean values and standard errors of the mean.

### 3.6 Children's Anxiety Before Surgery as Evaluated by the Medical Doctors

**Figure 4:**
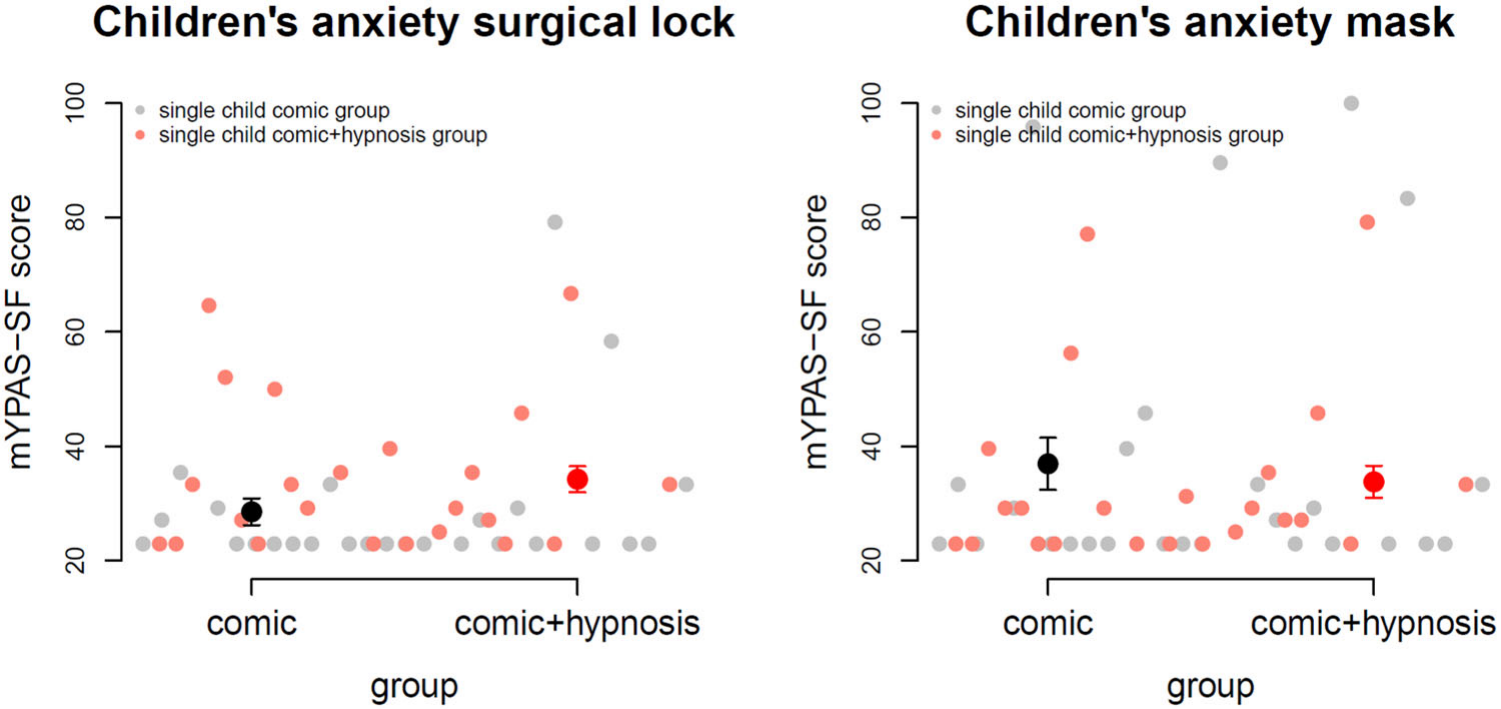
Children's anxiety before surgery, observed by medical doctors at the operating lock and when putting on the mask. Observed anxiety scores show low anxiety in children before surgery. mYPAS‐SF = Yale preoperative anxiety scale.

We measured children's anxiety with the Yale preoperative anxiety scale mYPAS‐SF (original YPAS [25]; modified short form [26]), where medical doctors rate children's behavior on two occasions: at the operating lock and when children got the mask on. Possible values range from 20 for very low anxiety and 100 for very high anxiety. We found no significant group differences at both time‐points (operating lock: *p* = 0.13; mask: *p* = 0.60). There was also no significant difference between time‐points (*p *= 0.24). Observed anxiety was generally very low as rated by medical doctors with the mean mYPAS‐SF scores of 33.3. Figure 4 shows scatter plots, mean values, and standard errors of the mean for both groups and time‐points.

### 3.7 Children's Pain After Surgery

**Figure 5:**
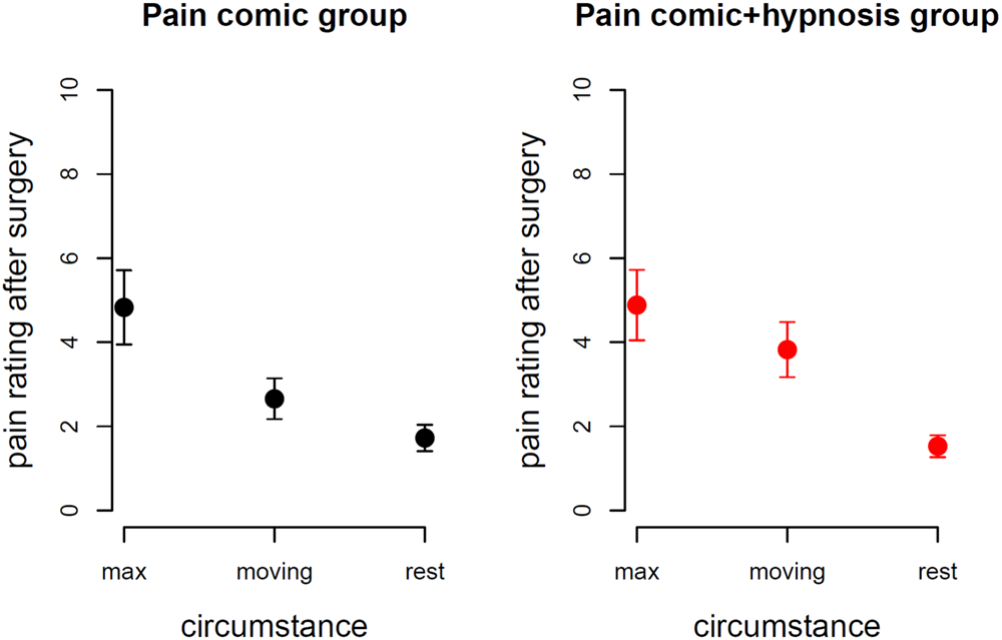
Children's pain ratings 1 day after surgery on a visual analogue scale. Low values indicate low pain. We did not find significant group differences, but the expected difference between maximal pain, pain when moving, and pain during rest.

The day after surgery, we called the parents and asked them about their child's pain state using the QUIPS‐infant pain questionnaire [22]. In the QUIPS‐infant, children rate their pain using the validated faces scale according to Hicks et al. [23] and a standardized procedural guide. The pain scale ranged from 0 for no pain to 10 for very much pain. We found no significant differences between groups for all three pain intensity circumstances (maximal pain: *p* = 0.95; pain when moving (e.g., swallowing): *p* = 0.08; pain during rest: *p* = 0.73). We found that maximal pain ratings were significantly higher than moving pain ratings: *t*(62) = 5.2, *p* < 0.001 and significantly higher than pain ratings during rest: *t*(62) = 9.5, *p* < 0.001. Moving pain ratings were significantly higher than pain ratings during rest: *t*(62) = 6.5, *p*< 0.001. Mean ratings for the three circumstances show medium pain for maximum ratings (4.9), moderate pain when moving (3.3), and low pain during rest (1.6). Figure 5 shows all mean ratings with standard errors of the mean for both groups and all three circumstances.

### 3.8 Postoperative Well‐Being of Children

During the follow‐up phone call, we also assessed other variables of children's well‐being in a simple yes/no format. The pain when coughing or when taking a deep breath did not differ significantly between groups (*p* = 0.96). The percentage of pain occurring when coughing or taking a deep breath was 24%. We also asked children if they woke up during the night because of pain. There was no significant difference between groups (*p* = 0.96), and 24% of children reported that they woke up because of the pain. We also asked children if they wished for more medication against pain. There were no significant group differences (*p* = 0.61), and 13% wished for more pain medication. The next question was if children felt tired. There was no significant group difference (*p* = 0.83), and 47% of children reported that they felt tired 1 day after surgery. The final question asked children if they felt sick since the surgery. There were no significant group differences in postoperative nausea (*p* = 0.51), and 10% of children reported that they felt sick since the surgery the day before.

In the post‐surgery phone call, we also gave the opportunity of open comments on the study. One mother told us that their child asked about Manchu the monkey immediately after surgery and then found a stuffed monkey at home that it now plays with very often since the surgery. The stuffed Manchu helps the child to overcome negative feelings. One father told us that he thinks the hypnosis audio intervention helped the mother and the child in the same way to reduce their preoperative anxiety. One child wanted to hear the hypnosis audio intervention again after the surgery was over. We collected the open commentaries and made them available via the Zenodo link https://doi.org/10.5281/zenodo.10265951.

## 4 Discussion

The goal of our study was to test if a hypnosis audio intervention reduces perioperative anxiety and postoperative discomfort of 3–6‐year‐old children undergoing surgery more than a narcosis comic that is part of our standard treatment at the Jena University Hospital. We found that there was no additional benefit of the hypnosis audio intervention. All children had very low perioperative anxiety and postoperative discomfort. Also, parents' preoperative anxiety ratings were moderate in all parents.

Preoperative anxiety is very common for children with incidence rates up to 75% [1], so it is remarkable that we found very low anxiety ratings in our study. We had both self‐ratings and observed ratings of anxiety in our study: we asked children themselves to rate their discomfort with a face rating scale, and medical doctors rated children's anxiety on the day of the surgery. Both scales showed very low preoperative anxiety. As a consequence of our results, it is possible to reduce or even omit premedication with midazolam as preoperative anxiety was so low in our sample. Previous results show that hypnosis is as effective as midazolam to reduce preoperative anxiety [19]. Midazolam can produce negative side effects like anterograde amnesia while implicit memory remains intact [29] and adverse postoperative behavior [30]. Our hypnosis intervention might provide an alternative to medication for children before surgeries to reduce their anxiety.

Postoperative pain ratings were moderate regarding the surgery the day before. The expected difference between maximal pain, pain during movements, and pain at rest show that children were able to differentiate their experienced pain. The observed pain ratings 1 day after surgery in our sample are comparable to other studies using the QUIPS‐infant in children undergoing surgery at the Jena University Hospital [31, 32].

Due to our study protocol, parents and their children only got our material 1 day before surgery. It might be beneficial to give parents and children more time to prepare with the narcosis comic and the hypnosis audio intervention. That might explain the dropout of children in the comic+hypnosis group who failed to listen to the 18 min hypnosis audio intervention at least once without breaks before surgery.

In our study, we did not have a group of children that did neither get the narcosis comic nor the hypnosis audio intervention. As we have already shown that the narcosis comic reduces preoperative anxiety (Doctoral Thesis by Dr. Rebecca Rädel), we did not want to keep this narcosis comic from a group of children to avoid possible disadvantages from taking part in the study. The consequence is that we cannot compare preoperative anxiety of our sample with a group that did not receive any nonmedical intervention.

In the Jena University Hospital, we now use a combination of wall posters, the comics, and the hypnosis audio flyer, all showing Manchu, the little monkey. The goal is to improve child‐friendly care in otolaryngology. The contents of our interventions help children and their parents to understand all necessary steps of the surgery and reduce perioperative anxiety. It is possible to include further attempts like mobile applications [33] and combine them with our hypnosis audio intervention. The goal is to produce positive mental images like a little monkey on a space journey having a great time during its adventure in the hospital. As some children and their parents reported, they even look forward to the surgery when they perceive it as a desirable adventure. A positive first healthcare experience as a child can have a powerful effect on children's lives later on as they expect positive experiences and perceive themselves as active and able to cope with the challenges that might occur [34].

We designed our material for 3–6‐year‐old children and their parents to educate them about the processes in the hospital and providing them positive mental evaluations of all procedures. Our comic and hypnosis interventions do not need additional personnel resources. Our data show that both the group of children that received the narcosis comic and the group of children receiving the narcosis comic and the hypnosis audio intervention had low perioperative anxiety and low discomfort ratings after surgery. We conclude that both our narcosis comic and the hypnosis audio intervention are contributing to better treatment of children undergoing surgery.

## Author Contributions

**Barbara Schmidt:** conceptualization, data curation, formal analysis, funding acquisition, investigation, methodology, project administration, resources, software, supervision, validation, visualization, writing–original draft, writing–review and editing. **Claudia Thomas:** conceptualization, data curation, methodology, writing–review and editing. **Antje Göttermann:** methodology, project administration, writing–review and editing. **Winfried Meißner:** conceptualization, methodology, resources, writing–review and editing. **Katharina Geißler:** conceptualization, data curation, methodology, project administration, resources, writing–review and editing. **Orlando Guntinas‐Lichius:** conceptualization, methodology, supervision, writing–review and editing. **Anne Schirrmeister:** conceptualization, data curation, methodology, writing–review and editing. All authors have read and approved the final version of the manuscript.

## Conflicts of Interest

The authors declare no conflicts of interest.

## Transparency Statement

The lead author, Barbara Schmidt, affirms that this manuscript is an honest, accurate, and transparent account of the study being reported, that no important aspects of the study have been omitted, and that any discrepancies from the study as planned (and, if relevant, registered) have been explained.

## Supporting information

Supporting information.

## Data Availability

Barbara Schmidt had full access to all of the data in this study and takes complete responsibility for the integrity of the data and the accuracy of the data analysis. We made all anonymized data, analysis scripts, the hypnosis audio intervention, and other study materials available via Zenodo: https://doi.org/10.5281/zenodo.10265951.
