# Supplementary material for: Reducing Perioperative Anxiety and Postoperative Discomfort in Children With Hypnosis Before Tonsillotomy and Adenoidectomy: A Prospective Randomized Trial
Source: Health Sci Rep. 2025 Mar 9;8(3):e70484. doi: 10.1002/hsr2.70484 (PMC11891016; doi:10.1002/hsr2.70484)
Supplement: Supplementary file 1 — Supporting information. [file HSR2-8-e70484-s001.docx]

Supplemental Material for HypnoChild manuscript

**Description of narcosis comic**

The comic describes all successive events that occur on the day of children’s surgery. The main character in the narcosis comic is a little monkey called Manchu. This comic was developed by the coauthors Dr. Anne Schirrmeister and Dr. Claudia Thomas and drawn by the graphic artist Sandra Bach (<https://www.sandruschka.de/>). The comic is available via this link: <https://www.uniklinikum-jena.de/kai/Patienten+und+Besucher/Narkose/Kinder/Narkosecomic.html>. The little monkey Manchu is also present via wall stickers in the premedication unit and the surgery facilities of the Jena University Hospital. All children received the narcosis comic one day before the surgery at the premedication unit of the Jena University Hospital as a small printed book with 25 pages. In the following, we summarize the content of the narcosis comic. First, Manchu the monkey introduces himself. He visits children before and after narcosis. Children learn that they will not have breakfast on the surgery day, but that they can drink until two hours before surgery. The monkey Manchu explains that there will be a sweet drink to calm children down and a magic plaster on their skin so that they will not feel the pinprick afterwards. Children learn that their parents will wait outside the surgery facilities. On a central page, the narcosis procedure is explained with all necessary parts. There are stickers on children’s chest to record their heart activity, a finger clip drawing colored waves on the screen, a butterfly on their skin that can cause a little ache but because of the magic plaster, they might not realize it at all. Also, they will wear a device to measure their heart power via a tight pressure on their arm. To describe the anesthesia procedures, children learn that there will be an astronaut mask on their face with dream air so they can inflate a balloon while the white dream milk is tickling their arm. The result is that the child in the comic gets very tired and falls asleep. The little monkey Manchu then suggests a funny dream where the child and monkeys play in the jungle with other happy animals. Then, the comic describes how it will be to wake up after surgery is over with a little ache in their throat and their parents around who offer the child ice cream. Finally, children learn that they will be back to the children’s station of the hospital meeting other children and talk about their experiences at the hospital.

**Description of hypnosis audio intervention**

We developed the hypnosis audio intervention with similar contents as the narcosis comic. The protagonist Manchu the monkey himself talks to the child. The audio intervention is available here https://manchu.uni-jena.de/Home/Index?code=8bfa9aea-a744-447f-9ab1-0c9edb364915 and takes 18 minutes. Children in the experimental group got a flyer with a QR code leading them to the hypnosis audio intervention. We developed a way to check if children listened at least once to the whole hypnosis audio intervention as we personalized the QR code for every child. In the following, we summarize the content of the hypnosis audio intervention. First, Manchu the little monkey introduces himself and tells the child that he lives in the hospital where they have already seen each other before. When Manchu saw the child in the hospital for the first time, he realized that this child is strong and brave. That made him very happy. Manchu says that he comes from another planet, the dream planet. That is a safe place where everything is beautiful. His friends and family live there, and you can have a great time on this planet playing and singing. If the child wants to know how to get to the dream planet, he offers to explain. Manchu says that there is a way to get to the dream planet via the start ramp in the hospital. To get to the dream planet, it is necessary to be strong and brave like the child. It is possible that the child feels nervous, which is normal. Then, Manchu describes how it will be in the hospital on the day of the surgery. All people wearing green suits are there to help the child to get to the dream planet. The child gets its own astronaut’s suit. There will be a sweet drink and a magic plaster on its skin. It might feel like at the airport flying to a holiday destination for the first time, a little nervous but looking forward to the holiday. Manchu the monkey describes the bright stars on the way to the start ramp. Then, there is a person in a green suit taking care of the child’s parents who will wait for it and are proud that the child is so brave and adventurous. Now, it is time to enter the dream rocket. The rocket might look like a bed, but it has the power to get the child to the dream planet. Now, the child enters the last station before the space journey begins. Colored stickers on children’s skin make it curious about the colors on the dream planet. The power measure on its arm shows all people around how strong the child is. Everything is ready for take-off now. Manchu the monkey jumps in the dream rocket together with the child. Together, they are looking forward to their adventures on the dream planet. Takeoff feels strange as it is the first time, there might be a tickling feeling in the body and the child gets tired. That is a sign that the dream planet is very close. The child lands successfully on the dream planet and has a wonderful time with Manchu’s monkey family. Everyone is happy to see the child, singing and dancing around. They all get friends and play together. They are laughing so loud that many other animals are joining and everyone is dancing. Everyone on the dream planet takes care of the child. After a while, the child is ready to fly back to the hospital. It says goodbye to all its new friends and is happy and proud about its adventure. Back in the hospital, the child awakes. There is plenty of time to arrive and wake up. The parents are already there. The child will be hungry and can eat. Then, the child tells its parents about its adventures on the dream planet. The parents are happy and very proud about their strong and brave child. Also, Manchu the monkey is proud and tells the child that he and his family like the child very much and that the child can come back anytime. The only thing it has to do is to think about the dream planet.
